# Single-cell transcriptomic analyses of mouse idh1 mutant growth plate chondrocytes reveal distinct cell populations responsible for longitudinal growth and enchondroma formation

**DOI:** 10.21203/rs.3.rs-4451086/v1

**Published:** 2024-06-04

**Authors:** Vijitha Puviindran, Eijiro Shimada, Zeyu Huang, Xinyi Ma, Ga I Ban, Yu Xiang, Hongyuan Zhang, Jianhong Ou, Xiaolin Wei, Makoto Nakagawa, John Martin, Yarui Diao, Benjamin A. Alman

**Affiliations:** Duke University School of Medicine; Duke University School of Medicine; Duke University School of Medicine; Duke University School of Medicine; Duke University School of Medicine; Duke University School of Medicine; Duke University School of Medicine; Duke University School of Medicine; Duke University School of Medicine; Duke University School of Medicine; Duke University School of Medicine; Duke University School of Medicine; Duke University School of Medicine

## Abstract

Enchondromas are a common tumor in bone that can occur as multiple lesions in enchondromatosis, which is associated with deformity of the effected bone. These lesions harbor mutations in *IDH* and driving expression of a mutant *Idh1* in Col2 expressing cells in mice causes an enchondromatosis phenotype. In this study we compared growth plates from E18.5 mice expressing a mutant *Idh1* with control littermates using single cell RNA sequencing. Data from Col2 expressing cells were analyzed using UMAP and RNA pseudo-time analyses. A unique cluster of cells was identified in the mutant growth plates that expressed genes known to be upregulated in enchondromas. There was also a cluster of cells that was underrepresented in the mutant growth plates that expressed genes known to be important in longitudinal bone growth. Immunofluorescence showed that the genes from the unique cluster identified in the mutant growth plates were expressed in multiple growth plate anatomic zones, and pseudo-time analysis also suggested these cells could arise from multiple growth plate chondrocyte subpopulations. This data identifies subpopulations of cells in control and mutant growth plates, and supports the notion that a mutant *Idh1* alters the subpopulations of growth plate chondrocytes, resulting a subpopulation of cells that become enchondromas at the expense of other populations that contribute to longitudinal growth.

## INTRODUCTION

Enchondromas are one of the most common benign tumors occurring in bone, occurring in about 3% of the population[1, 2]. They are composed of cells derived from growth plate chondrocytes and can occur as solitary lesions or as multiple lesions in enchondromatosis syndromes. Enchondromas can progress to malignant chondrosarcomas, an occurrence that is more common in multiple enchondromatosis. Somatic mutations in *IDH1* and *IDH2*, the genes encoding isocitrate dehydrogenase proteins are present in the majority of enchondromas and in at least half of chondrosarcomas[3–5]. The *IDH* genes encode for enzymes that convert isocitrate to alpha-ketoglutarate (a-KG), a component in the citric acid cycle and a metabolic fuel. IDH 1 and 2 reside in the cytoplasm and mitochondria, respectively. The mutant IDH found in enchondromas, and chondrosarcoma produces D-2-hydroxyglutarate (D-2-HG)[6]. IDH1 and IDH2 mutations in tumors are heterozygous, because their wild-type activities are essential for cellular respiration and metabolic function[3, 4, 7, 8]. D-2-HG is sometimes called an “oncometabolite”[6], and is shown to have epigenetic effects related to histone and DNA hypermethylation; stabilize Hypoxia Induced Factor one alpha (Hif-1α) protein; impair cellular differentiation; increase cell proliferation; and increase the expression of stem cell markers[9–12]. However, the effect of mutant IDH is cell type dependent [13] and its role in chondrocytes is not completely elucidated.

Mice expressing the Idh1-R132Q mutation driven by regulatory elements of type 2 collagen (*Col2a1-Cre; ldh1*^*LSL− R132Q L/WT*^) show a delay in growth plate terminal differentiation but exhibit perinatal lethality. A temporally regulated mouse, *Col2a1-Cre/ERT2; ldh1*^*LSL− R132Q L/WT*^, in which Cre expression is induced in Col2 expressing cells by tamoxifen after weaning, developed multiple enchondroma lesions. Thus, an Idh somatic mutation gives rise to growth-plate cells that persist in the bone as enchondromas, failing to undergo normal differentiation [5].

While bulk expression profiling showed differences between chondrocytes expressing a mutant and wild type Idh[14, 15], such analyses cannot identify changes in subpopulations of cells. To determine how a mutant Idh might change the behavior of specific subpopulations of growth plate cells, we undertook single cell RNA sequencing to compare growth plates from *Col2a1-Cre; ldh1*^*LSL− R132Q L/WT*^ animals with those from control littermates. Because the growth plates and enchondromas that develop in *Col2a1-Cre/ERT2; ldh1*^*LSL− R132Q L/WT*^ mice contain very few cells, it was not technically feasible to use these animals for single cell RNA analysis.

## RESULTS

### Single-cell RNA analysis identified eight chondrocyte subtypes in the embryonic growth plate.

**Figure 1:**
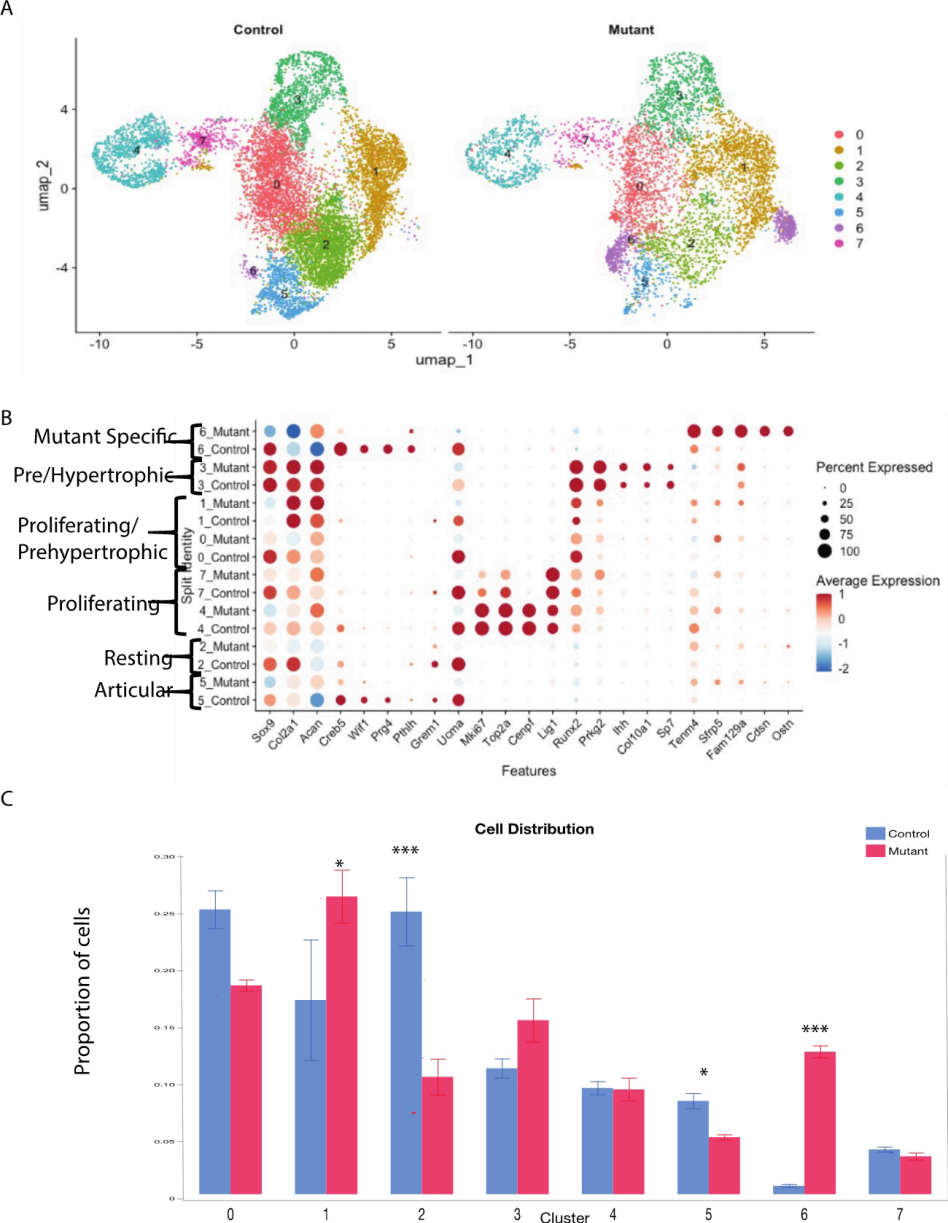
ScRNA-Seq analysis of growth plates expressing mutant Idh1 and littermate controls expressing wild-type Idh1. A) UMAP visualization of cell clusters from scRNA sequence analysis of Control (left) and Mutant (right) shows 7 clusters; B) Dot plot showing the expression of selected top markers for each cluster and cell type annotation. Dot size represents the percentage of cells expressing a specific marker, while the red (higher) and blue(lower) colors indicates the average expression level for that gene, in that cluster; C) Bar graph showing the percentage of cells in each cluster from Control and Mutant. Statistical analysis was performed using 2-way repeated measure ANOVA; *P <0.05, ***P <0.0001. n = 3.

To investigate how the expression of a mutant *Idh1* alters cell populations in growth plate chondrocytes in a way that could cause the development of enchondromas, we performed single-cell RNA sequencing (scRNA-seq) analysis using embryonic growth plate chondrocytes from E18.5 *Col2a1-Cre; ldh1*^*LSL− R132Q* L/WT^ (Idh1 mutant knock-in) and *ldh1*^*LSL− R132Q L/WT*^ (Cre negative) controls. Cells from three animals were used for each genotype. 6061 cells from mice expressing the mutant Idh1 and 10562 cells from control animals were analyzed (supplementary materials, Table S1). The Uniform Manifold Approximation and Projection (UMAP) dimensionality reduction method was used to visualize similar cells together in two-dimensional space, and this clustering via the Louvain method was performed at a resolution of 0.28. Chondrocytes express genes that function to produce a specialized extracellular matrix including collagen type two and glycosaminoglycan [16]. Genes expressed by all chondrocytes, *Col2a1*, *Acan* and *Sox9* showed abundant expression in all the cells from control or mutant animals in this analysis (Fig. 1, Supplementary Fig. 2). Growth plate chondrocytes undergo a tightly regulated differentiation pathway including low proliferating chondrocytes, also termed resting chondrocytes and articular chondrocytes. Adjacent to the resting cells, proliferating chondrocytes, sometimes termed as column-forming flat chondrocytes are present, which differentiate into pre-hypertrophic chondrocytes which have a nominal proliferation rate. Pre-hypertrophic chondrocytes undergo a rapid volumetric increase to differentiate into hypertrophic chondrocytes (HC) [17–23]. Analysis revealed eight clusters within the chondrocyte population (Fig. 1A and Supplementary Fig. 1A and B). Growth plate chondrocyte subtype types were annotated based on the marker genes expressed (Supplementary Figs. 2 and 3),

Wnt inhibitory factor 1 (*Wif1*) and *Creb5*, genes expressed by articular chondrocytes [24], and this cluster was annotated as such. Cluster 2 was highly enriched for expression of *Grem1* along with cartilage matrix associated protein (*Ucma*), genes expressed in resting chondrocytes [25]. Cluster 4 and 7 show increased expression of cell cycle genes, such as *Mki67, Top2a, Cenpf* and *Lig1* and are designated as proliferating chondrocytes [26]. Runt-related transcription factor 2 (*Runx2*) induces chondrocyte maturation, enhances chondrocyte proliferation through Ihh induction and is expressed by proliferating and prehypertrophic chondrocytes [18], and was highly expressed in Clusters 0 and 1. Cluster 3 was enriched in Protein Kinase CGMP-Dependent 2 (*Prkg2*) which is required for the proliferative to hypertrophic transition of the growth plate chondrocytes [27], Indian hedgehog (*Ihh*) [28], Collagen type X (*Col10a1*) [29] and Sp7. These genes are expressed in both prehypertrophic chondrocytes and preosteoblasts, consistent with the potential for differentiated growth plate chondrocytes to become osteoblasts [30]. Markers of terminal hypertrophic cells (markers such as *Mmp13* [31]) were not present in our data set (supplementary Fig. 2), consistent with hypertrophic chondrocytes being beyond the volume of conventional single cell sequencing approaches[32].

### A distinct chondrocyte subpopulation is identified in Idh1 mutant cells:

**Figure 2:**
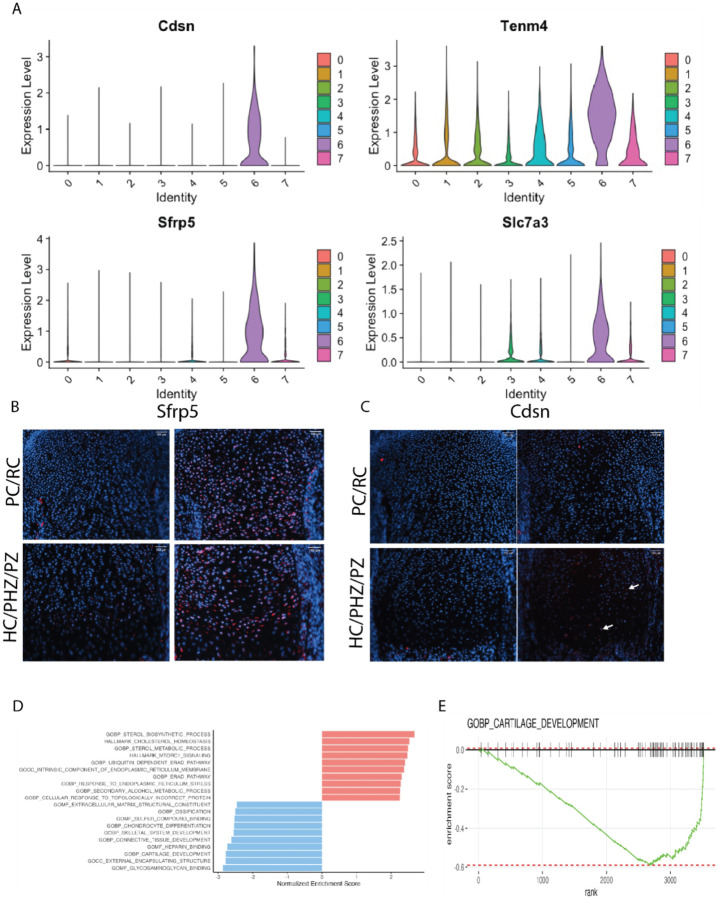
ScRNA-Seq analysis reveals distinct cluster contributed by the mutant Idh1 expressing chondrocytes: A) Violin plots showing the expression of top feature genes in the distinct cluster 6 - Cdsn, Tenm4, Sfrp5, and Slc7a3. B&C) Immunofluorescence for Sfrp5 and Cdsn from representative mutant and control growth plates; B) Sfrp5 protein expression was detected in resting (RC), proliferating (PC) and prehypertrophic (pHC) chondrocytes in the mutant while it was restricted to few cells in the PC and pHC chondrocytes in the control; C) Cdsn protein was detected in PC and pHC zones (white arrows) in Idh1 mutant and absent in the control growth plates; D) Gene Set Enrichment Analysis (GSEA) of cluster 6 showing upregulated (shown in red) and downregulated (shown in blue) pathways; E) GSEA plot showing that cartilage development is downregulated in cluster 6.

Cluster 6 was contributed primarily by *Idh1* mutant cells and was found in all the biological replicates from mutant animals (Supplementary Fig. 1A). About 12% of Idh1 mutant cells and less than 1% of control cells contributed to this cluster (p < 0.0001, Fig. 1B). This cluster was enriched in Teneurin-4 (*Tenm4*) [33], Corneodesmosin (*Cdsn*) [34], Secreted Frizzled Related Protein 5 (*Sfrp5*) [35] and solute carrier family 7 member 3 (*Slc7a3*) (Fig. 2A and gene profiles in supplementary data). *Tenm4* is a transmembrane protein that can suppresses chondrogenic differentiation [33]. *Sfrp5* can inhibit the Wnt signaling pathway, and it is expressed in proliferating and pre-hypertrophic growth plate cells [35]. Using immunofluorescence, we found that Sfrp5 protein expression was restricted to the proliferating and prehypertrophic in control samples, however the number of cells expressing and region of expression was expanded in the mutant samples (Fig. 2B). Cdsn is an extracellular glycoprotein essential for maintaining the skin barrier in adult skin and normal hair follicle formation that is not characteristically expressed in chondrocytes [36], but was detected in the mutant samples (Fig. 2C). The amino acid transporter protein solute carrier family 7 member 3 (*Slc7a3*) is a sodium-independent cationic amino acid transporter that mediates the uptake of the cationic amino acids arginine, lysine and ornithine in a sodium-independent manner, and is upregulated due to glutamine deprivation [37]. Chondrocytes with an *Idh1* mutation are known to utilize glutamine as a cell energy source [15] and *Slc7a3* upregulation is consistent with the notion that it these cells utilize glutamine at a high rate.

**Figure 3:**
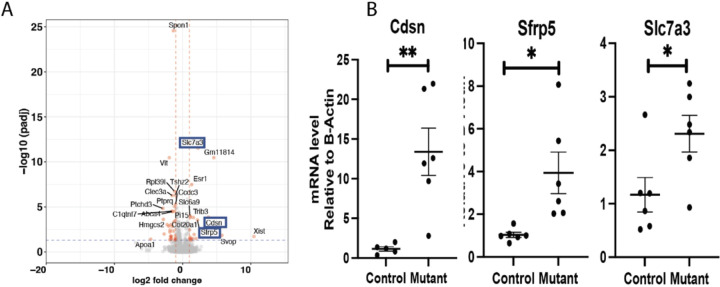
Bulk RNA sequencing from micro-dissected growth plates detects upregulation of genes in cluster 6. A) Volcano plot showing differential expression between mutant and control growth plates: Sfrp5, Cdsn and Slc7a3 were significantly upregulated, marked in blue box; B) Realtime quantitative RT-PCR confirming upregulated gene expression of Sfrp5, Cdsn and Slc7a3.

*Matn1, Col9a1, Cnmd*, and *Matn3* were the most downregulated genes in Cluster 6. These genes encode for proteins important in cell-matrix interaction [38]. Gene Set Enrichment Analysis (GSEA) for cluster 6 genes, showed several downregulated pathways in this cluster (Fig. 2D and E) including ones involved in cartilage development extracellular matrix structural constituents, ossification, chondrocyte differentiation, and skeletal system development. There was also upregulation of genes involved in the cholesterol homeostasis pathway, sterol metabolic process, and mTORC1signaling pathways (Supplementary Fig. 4). Some of genes identified as highly expressed in cluster 6, such as *Slc7a3, Cdsn* and *Sfrp5* were also identified as differentially regulated in bulk RNA sequencing analysis (Fig. 3A). A gene that is highly expressed in cell subpopulation can be identified in bulk sequencing if it is not expressed in other cell subpopulations. This finding is constant with the notion that these genes are primarily expressed in this unique cluster.

### Two cell populations, cluster 2 and 5, were underrepresented in Idh1 mutant animals:

**Figure 4:**
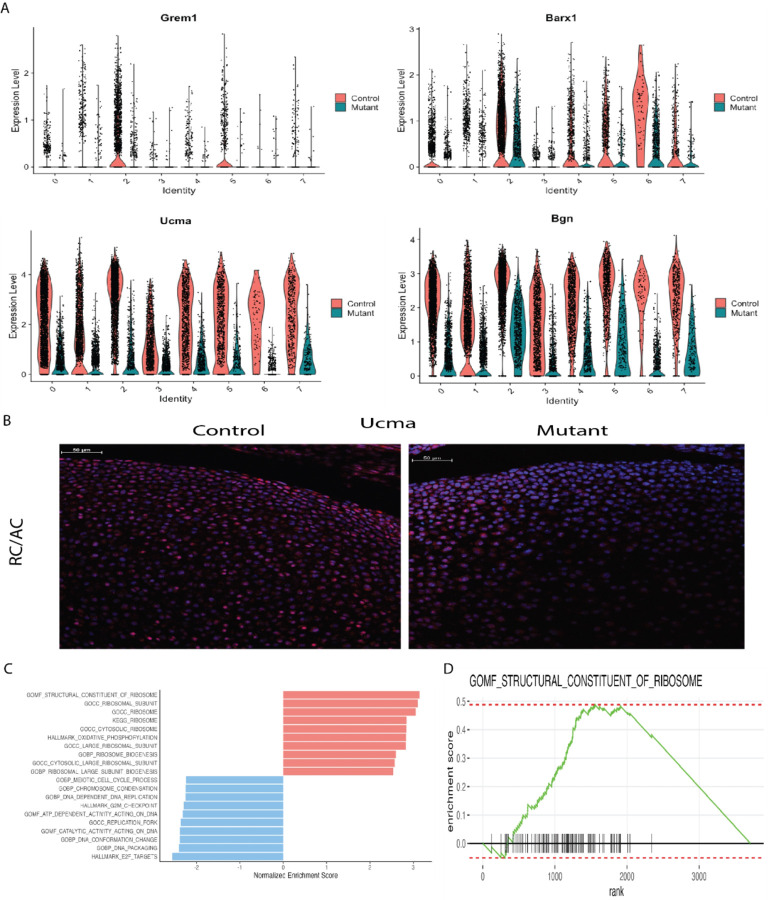
Cell populations (cluster 2, resting chondrocytes) that are underrepresented in the mutant growth plates. A) Violin plots showing the expression of top genes in cluster 2, Grem1, Barx1, Ucma, and Bgn. B) Immunofluorescence for Ucma protein – it’s expression was detected in articular chondrocytes and resting chondrocytes in the control growth plate while in the mutant, Ucma expression is almost absent in these regions; C) GSEA of cluster 2 showing upregulated (shown in red) and downregulated (shown in blue) pathways; D) GSEA plot showing that molecular function of structural constituent of ribosome pathway is upregulated in this cluster 2.

**Figure 5:**
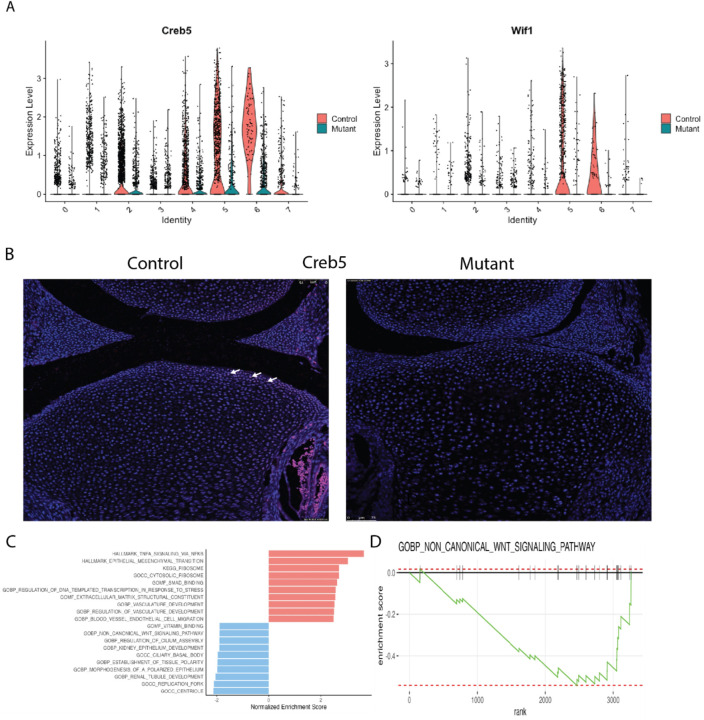
Cell populations (cluster 5, articular chondrocytes) that are underrepresented in the mutant growth plates. A) Violin plots showing the expression of top genes in cluster 5, Creb5 and Wif1; B) Immunofluorescence for Creb5 protein – its expression was detected in articular chondrocytes in the control growth plate (white arrows) while in the mutant, Creb5 expression is almost absent; C) GSEA of cluster 5 showing upregulated (shown in red) and downregulated (shown in blue) pathways. D) GSEA plot showing that in this cluster 5, biological process of non-canonical WNT signaling pathway is downregulated.

In addition to identifying a cluster of cells uniquely present in the Idh1 mutant animals, we identified two cell populations which were composed of s fewer Idh1 mutant cells. Cluster 2 is composed of about 10% from mutant, while 24% from control cells (supplementary Table S2). This cluster expresses *Grem1*, *Barx1*, *Ucma* and *Bgn* (Fig. 4A). *Ucma* is normally expressed in differentiated chondrocytes, [20, 39, 40], and immunofluorescence verified that mutant growth plate has decreased Ucma expression compared to the controls (Fig. 4B). The GSEA analysis of this cluster identifies pathways which upregulated in ribosome biogenesis, protein biosynthetic processes as well as peptide metabolic process (Fig. 4C, 4D and supplementary Fig. 5A). Chondrocytes require high translational capacity to meet the demands of proliferation, matrix production, and differentiation [41]. Thus, this cluster may play a role in longitudinal bone growth, and growth plates lacking cells in this subpopulation may be responsible for the observed short limb phenotype. Cluster 5 also showed a significant decrease in the mutant cells (Fig. 1C and 5A). This cluster expressed genes characteristic of articular chondrocytes, and immunofluorescence for Creb5, a gene expressed in this cluster shows very few chondrocytes staining in the mutants compared to the control (Fig. 5B).

#### Pseudotime analysis shows differences between mutant and control growth plates.

**Figure 6:**
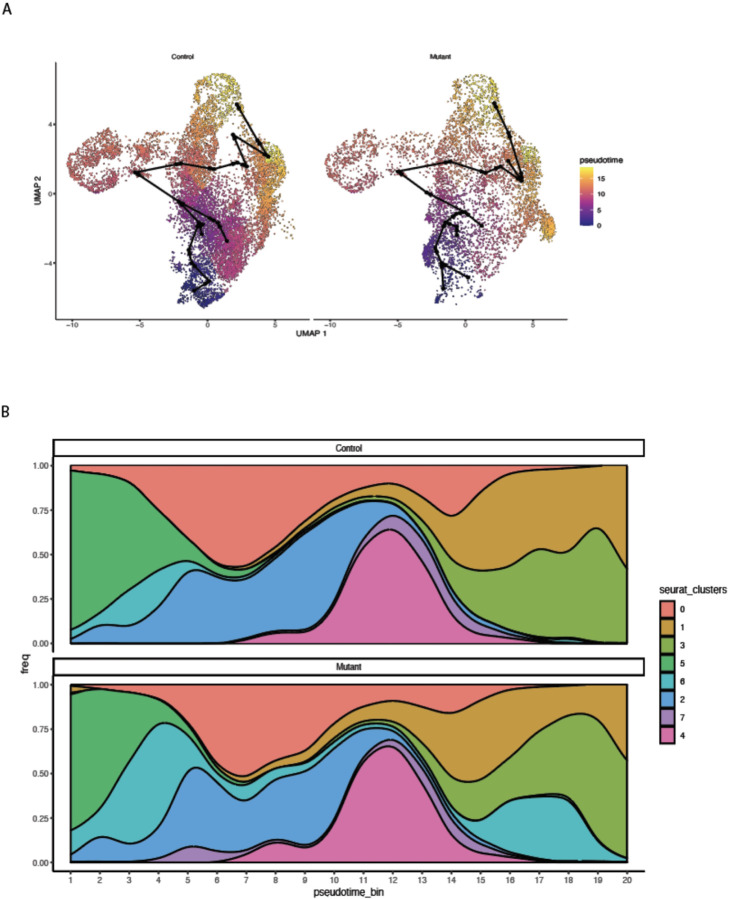
Pseudotime analysis. A) The UMAP plots show pseudotime trajectories of subtypes of chondrocytes. The slingshot lineage structure, which is based on the relative positions of 20 bins of monocle3 pseudotime ordering, is illustrated by black lines with arrows. B) Population graph showing the cells in pseudotime bins arranged from left (early) to right (late), in 1–20 bins.

To investigate additional differences between control and Idh1 mutant chondrocytes that might be identified using single cell analysis, cells were ordered using pseudotime analysis in an unsupervised manner. As anticipated, cluster 5 (articular chondrocytes) and cluster 2 (resting chondrocytes) map to early stages, while proliferating and prehypertrophic chondrocytes were from middle to late stages in the pseudotime scale, respectively (Fig. 6A). The cluster present primarily in mutant animals (cluster 6) was split into early and mid-late scales suggesting that this population is present at multiple stages in growth plate development, and that it is comprised of cells that remain in a less differentiated stage (Fig. 6B).

## DISCUSSION

The concept that enchondromas derive from growth plate cells that fail to undergo differentiation during longitudinal growth is supported by the anatomic finding that enchondromas exist adjacent to growth plates, and by data from mice in which enchondromas develop when genetic alterations identified in human tumors are driven in type two collagen expressing cells. Our data from single cell analysis is consistent with this notion and suggests that that there is a unique subpopulation of chondrocytes in the growth plates from mice expressing a mutant *Idh1*.

The unique cluster identified in the *Idh1* mutants expresses genes known to be upregulated and downregulated in enchondromas [42]. Immunofluorescence for the proteins corresponding to the genes expressed in this population are not anatomically located in a single location on the growth plate but distributed throughout several zones. Pseudotime analsysis was also consistent with this population being present at multiple stages in growth plate development, likely or an early developmental origin. The subpopulation of cells underrepresented in the mutant growth plate expresses genes that are known to play a role in longitudinal bone growth. It is possible that a shift from this subpopulation by mutant cells is to be responsible for the associated growth defectivity in limbs which contain multiple enchondromas. Cells in this subpopulation may play a more generalized role in longitudinal bone growth.

Single cell expression analysis is a powerful tool to identify populations of cells within a tissue and gene expression within individual cell subpopulations. This technique has been used to analyze a variety of tissues including tumors, developmental, and reparative processes. Here we used this approach to analyze growth plate cells expressing a mutation known to cause enchondromatosis. By comparing mutant and control cells, we identified a shift in cell subpopulations. This is consistent with the notion that enchondromas are formed by a shift in the fate of cells in the growth plate, leaving some cells to remain as enchondromas, and depleting some cells from populations responsible for longitudinal growth. Our data provides an atlas of gene expression analysis in mutant and control growth plate which can be used more generally to study bone development and growth.

Tumors can be made up of combinations of mutant and non-mutant cells [43], and our single cell data, along with the information from the localization of the genes expressed in the unique cluster found in mutant cells, is consistent with this possibility in enchondromas. Our data suggests that the study of specific cell populations may be more relevant to an understanding of specific pathologic processes. It also identifiedspecific cell subpopulations, and genes expressed in these subpopulations, as important in longitudinal long bone growth in general.

## Materials and Methods

### Animals and approval:

All animals were used according to the approved protocol by Institutional Animal Care and Use committee of Duke University. All experiments were performed in accordance with relevant guidelines and regulations. The study is reported in accordance with ARRIVE guidelines. The generation of Idh1^LSL/+^ [5] and Col2a1-Cre animals was previously reported [44].

### Isolation of growth plate chondrocytes from embryonic growth plate:

Using the Idh1R132Q lox-stop-lox (LSL) mouse, mutant Idh1 was expressed using Col2a1-Cre which will induce the expression in mouse chondrocytes. For single cell RNA seq, growth plate chondrocytes were harvested from the distal part of femur at E18.5 from Col2a1-Cre; Idh1R132Q LSL/+ and their litter mate controls expressing the wild type Idh1 followed by cell isolation using 2mg/ml Pronase (Roche) digestion at 37C shaker for 30 minutes with constant shaking, washed by PBS, and then digested by 3mg/ml Collagenase IV (Worthington) for 1 hour at 37C humidified incubator, washed with PBS, followed by 3mg/ml Collagenase IV digestion again in petri dish at 37C humidified incubator, and filtered using 45um cell strainer. The live cells were sorted and loaded on the 10x Genomics Chromium using the Chromium Single Cell 3’ Reagent V3 Kit and the sequencing libraries were constructed following the user guide.

### scRNA-seq data pre-processing for 3’-end transcripts:

Cell Ranger version V3.0.2 (10x Genomics) was used to process raw sequencing data before subsequent analyses. These RNA sequencing reads were then aligned against refdata-cellranger-mm10–3.0.0 transcriptome to quantify the expression of transcripts in each cell to create feature-barcode matrices. The analyses of processed scRNA-seq data were carried out in R version 4.1.0 using the Seurat v4 for downstream analysis [45] &[46]. This data is deposited in the Gene Expression Omnibus (GEO) under accession number GSE201606.

In Seurat, the data was first normalized to a log scale after basic filtering for minimum gene and cell observance frequency cut-offs (http://satijalab.org/seurat). Initial quality control filtering metrics were applied to each sample dataset such to avoid empty and dying cells (i.e. n_Count_RNA > = 1000; nFeature_RNA > = 1000; log10Genespercount og10Genpercent.mt < 10 and min.cells = 3). Total of 10591 and 6069 cells from the controls and mutants were used in the following analyses, respectively. Principal components (PCs) were calculated using the most variably expressed genes and the first thirty PCs were carried forward for clustering and visualization. Cells were embedded into a K-nearest neighbor graph using the FindNeighbors function and grouped with the Louvain algorithm via the FindClusters function at resolutions of 0.3 to calculate the granularity of the clustering. The UMAP dimensionality reduction method was used to place similar cells together in two-dimensional space. Then, the cells were subset by Col2a1 expression > 2 from the integrated file, individual cell index was extracted and re-clustered at resolution 0.28. This led to total of 10562 and 6061 cells from the controls and mutants, respectively. The statistical analysis for percentage of cells distribution was performed using 2-way repeated measure ANOVA in JMP Pro 16 with installed Full Factorial Repeated Measures ANOVA Add-In. Cluster biomarkers were identified using the FindAllMarkers function, and differentially expressed genes between clusters were identified using the Wilcoxon test (p-value ≤ 0.05 was considered statistically significant).

### Single-cell differential gene expression analysis and GSEA for clusters of interest:

Single-cell differential gene expression analysis was conducted by Seurat “FindMarkers” function using “wilcox” (v1.14.0) [47] as the test method (R package). GSEA was implemented with fgsea [42] R package (v1.22.0) and the gene sets were imported from msigdbr R package (V7.5.1). Generally, the differential expressing genes with statistical significances (i.e., adjusted p-value < 0.10 and min.pct > 0.25 [i.e., minimum fraction of corresponding detected cells in either of the two populations]) were used for GSEA and the log2 fold changes were used as the pre-ranked scores. Four famous pathway/gene set databases were examined here (including Hallmark [43], KEGG [44], and Gene Ontology [45]). The pathways/gene sets with < 0.05 adjusted p-value were considered as significantly enriched pathways/sets.

### Pseudotime analysis:

Monocle 3 (v 1.2.9) was used for trajectory analysis [48]. The expression matrix was exported from the Seurat object and used as Monocle 3 input. Ordering of cells based on unsupervised learning and UMAP was used for dimensionality reduction. Then, pseudotime information was extracted from monocle3 data set. The pseudotime scale classified into 20 bins and number of cells were counted in each pseudotime bin. The population plot was generated using the function “ggstream” in Fig. 6B. Gene expression and pseudotime bins were extracted from the Seurat object, average gene expression was calculated, and cells were ordered in the scale of 0–50 pseudotime bins. The dot size indicates the number of cells in each pseudotime bin, and the blue-red color range indicates the expression level from low to high.

### Bulk RNAseq:

For Bulk RNAseq, E18.5 growth plate cartilages were harvested from distal part of femur and proximal part of tibia from Col2a1-Cre; Idh1R132Q LSL/+ and their litter mate controls. RNA was extracted using Norgen Biotech Single Cell RNA Purification Kit. Extracted total RNA quality and concentration was assessed on a 2100 Bioanalyzer (Agilent Technologies) and Qubit 2.0 (Thermo Fisher Scientific), respectively. Only extracts with RNA integrity number greater than 7 were processed for sequencing. RNA-seq libraries were prepared using the commercially available KAPA Stranded mRNA-Seq Kit. In brief, mRNA transcripts were first captured using magnetic oligo-dT beads, fragmented using heat and magnesium, and reverse transcribed using random priming. During the second-strand synthesis, the cDNA/RNA hybrid was converted into to double-stranded cDNA (dscDNA) and dUTP incorporated into the second cDNA strand, effectively marking the second strand. Illumina sequencing adapters were then ligated to the dscDNA fragments and amplified to produce the final RNA-seq library. The strand marked with dUTP was not amplified, allowing strand-specificity sequencing. Libraries were indexed using a 6–base pairs index, allowing for multiple libraries to be pooled and sequenced on the same sequencing lane on a HiSeq 4000 Illumina sequencing platform. Before pooling and sequencing, fragment length distribution and library quality were first assessed on a 2100 Bioanalyzer using the High Sensitivity DNA Kit (Agilent Technologies). All libraries were then pooled in equimolar ratio and sequenced. Multiplexing 8 libraries on one lane of an Illumina HiSeq 4000 flow cell yielded about 40 million 50 bp single end sequences per sample. Once generated, sequence data were demultiplexed and Fastq files generated using Bcl2Fastq conversion software provided by Illumina. This data is deposited in the Geo database accession number GSE201606.

### Bulk RNAseq Analysis:

RNA-seq reads were trimmed by Trim Galore (v 0.6.4) and mapped with STAR [49] (v2.6.1.d), with parameters –twopassMode Basic –runDirPerm All_RWX and supplying the Ensembl GRCm38 annotation to mouse genome (GRCm38). The mapped reads were counted using featureCounts [50] (v1.6.4). Bioconductor package DESeq2 [51] (v1.28.1) was employed to analyze differential expressions (DE) with litter and genotype information. Gene Ontology and KEGG enrichment tests were performed to analyze enriched biological processes by clusterProfiler [52] (v 3.16.1). The volcano plots were created by EnhancedVolcano (v 1.6.0). The coverage depth was normalized by deeptool [53] (v 3.1.3) using RKPM for RNA-seq. TPM values were quantified from Salmon [54] (v 1.2.1) quantification and summarized via tximport [7] (v 1.16.1). This data is deposited in the GEO database accession number GSE201606.

### Immunofluorescence:

E18.5 hindlimbs were fixed in 4%PFA overnight at 4C. The limbs were washed in PBS for 3 times and decalcified in 14%EDTA overnight at 4C. The limbs were washed again in PBS and incubated in 30% sucrose overnight at 4C and were embedded in Cryomatrix until the blocks became frozen in dry ice. The blocks were sectioned at 10um thickness for Immunofluorescence. The slides were brought to room temperature (RT) followed washes in PBS. Antigen retrieval was performed using 10mg/ml Proteinase K treatment for 10 minutes at room temperature followed by washes in PBS. The sections were blocked using 5% donkey serum and 0.3% Triton-X-100 in PBS for 1 hour at RT. Then the sections were diluted in the blocking serum and incubated overnight at 4C. The antibodies: anti-Ucma, Cat# PA520768, 1/200; For anti-CDSN antibody, Mybiosource, Cat# MBS713765, 1/50 dilution; anti-Sfrp5 antibody, Thermofisher, product #PA5–71770, 1/100 dilution; anti-Creb5 antibody, Thermofisher, Cat#PA5–65593). After washing with PBS, sections were incubated for 1 hour at room temperature with Alexa Fluor-594 secondary antibody (1:700, Jackson ImmunoResearch). The sections were washed with PBS before mounting with ProLong Glass Antifade Mountant with NucBlue Stain (Thermo Fisher Scientific, P36981), visualized by fluorescence microscopy (Axio Imager 2, Carl Zeiss).

#### Analysis of gene expression:

Total RNA was extracted from cells or tissues using RNAeasy mini kits (Qiagen) according to the manufacturer’s instructions. Total RNA was reverse transcribed in BioRad RT Reagent Kit to make cDNA. Quantitative real-time RT-PCR (BioRad) was performed using SYBR Premix (BioRad). Analysis of gene expression was performed using the ΔΔCt method. Data were normalized to expression of the beta-actin mRNA levels. Each experiment was performed in triplicates.

#### qRT-PCR Primer Sequence:

Sfrp5:FP- CCCTGGACAACGACCTCTGC; RP- CACAAAGTCACTGGAGCACATCTG
Cdsn: FP- CTGATGGCCGGTCTTATTCT; RP- GCTGTTGGAGCCAGTCTTTC
Slc7a3: FP – GGACTGTGTTATGCTGAATTTG; RP – CCAATGACGTAGGAGAGAATG

#### Study approval.

All the animal experiments were approved by Duke University’s Institutional Animal Care and Use Committee (IACUC).

## Data Availability

Single cell data and RNA sequencing is deposited in Geo database accession number GSE201606.
